# Controlling healthcare professionals: how human resource management influences job attitudes and operational efficiency

**DOI:** 10.1186/s12960-016-0149-0

**Published:** 2016-09-20

**Authors:** Julie Ann Cogin, Ju Li Ng, Ilro Lee

**Affiliations:** 1 Australian Graduate School of Management, University of New South Wales, Sydney, Australia; 2 Business School, University of New South Wales, Sydney, Australia; 3 Centre of Social Impact, University of New South Wales, Sydney, Australia

**Keywords:** HRM, Control, Commitment, Role theory, Healthcare management

## Abstract

**Background:**

We assess how human resource management (HRM) is implemented in Australian hospitals. Drawing on role theory, we consider the influence HRM has on job attitudes of healthcare staff and hospital operational efficiency.

**Methods:**

We adopt a qualitative research design across professional groups (physicians, nurses, and allied health staff) at multiple levels (executive, healthcare managers, and employee). A total of 34 interviews were carried out and analyzed using NVivo.

**Results:**

Findings revealed a predominance of a control-based approach to people management. Using Snell’s control framework (AMJ 35:292–327, 1992), we found that behavioral control was the principal form of control used to manage nurses, allied health workers, and junior doctors. We found a mix between behavior, output, and input controls as well as elements of commitment-based HRM to manage senior physicians. We observed low levels of investment in people and a concentration on transactional human resource (HR) activities which led to negative job attitudes such as low morale and frustration among healthcare professionals. While hospitals used rules to promote conformity with established procedures, the overuse and at times inappropriate use of behavior controls restricted healthcare managers’ ability to motivate and engage their staff.

**Conclusions:**

Excessive use of behavior control helped to realize short-term cost-cutting goals; however, this often led to operational inefficiencies. We suggest that hospitals reduce the profusion of behavior control and increase levels of input and output controls in the management of people. Poor perceptions of HR specialists and HR activities have resulted in HR being overlooked as a vehicle to address the strategic challenges required of health reform and to build an engaged workforce.

## Background

There is increasing evidence that human resource management (HRM) practices adopted by organizations can have a significant impact on firm performance [1]. The lesson from the literature is that effective human resource (HR) practices can positively contribute to organizations. Unfortunately, apart from a few studies [2–4], research that examines the linkage between HRM and firm performance has been transferred to hospital settings in a limited way despite the widely held belief that “management matters” in delivering quality healthcare outcomes. We posit that greater attention to the work that has been done in the HRM field could assist hospitals improve operational efficiency and job attitudes of staff. Such outcomes have consistently been cited as priorities for the healthcare sector which is faced with constant change and multiple challenges to deliver quality health services [5].

In response to these major challenges, and in an attempt to realize the benefits that effective HRM systems deliver, some scholars and healthcare reform committees have recommended that hospitals adopt a commitment-based approach to HRM [6]. This approach requires the development of mutual commitment between employer and employee, based on high levels of trust and empowerment [7].

Many of the calls for hospitals to change towards a commitment-based approach are driven by research which consistently suggests that the health sector adopts a control orientation to people management [6]. Typically a control approach is applied to jobs with low levels of skill variety, only provides basic levels of autonomy, and minimizes expenditure on people management. As a result, scholars argue that it is misaligned with stated healthcare reforms and constrains and demoralizes staff [8].

In this article, we explore the approach to people management implemented in Australian hospitals by using the control versus commitment typology. Drawing on role theory, we assess the influence HRM has on the job attitudes of staff and hospital operational efficiency. We do this by considering the roles played by different hospital actors (physicians, nurses, and allied health staff) at different levels (executives^1^, healthcare managers^2^, and employees^3^) in the working of HRM in an Australian context. We achieve these research aims via a qualitative research design.

### Commitment versus control

The mechanisms behind how commitment-based practices benefit organizations have been examined by numerous scholars. One view is that employees work harder due to their increased involvement and commitment which, in turn, comes from having more control and say in their work [9]. Others have found that commitment-based HRM practices enhance the social climate of the organization, creating higher levels of trust and cooperation among employees, which, in turn, drives performance [10].

Across the globe, healthcare reform committees have criticized the control-based people management approach employed in hospitals. This has provided the impetus for ongoing recommendations for hospitals to adopt a commitment-based approach to HRM [6, 11]. Despite wide criticism, there has been scant empirical research investigating the extent and types of control-based HRM in healthcare.

In this study, we undertake an exploratory investigation of the extent and type of control-based HRM employed in Australian hospitals by using Snell’s theory of control-based HRM [12]. According to Snell, managers can make the choice of a specific control mode, including behavior control, output control, or input control, to suit the situation. The type of controls used can produce positive outcomes; however, inappropriate forms of control can result in employee disengagement, high levels of absenteeism, turnover, and low morale [13].

#### Behavioral control

Behavioral control seeks to regulate employee actions through standardized jobs. This is achieved by supervisors structuring work and operating procedures and ensuring that employees adhere to established rules through managers closely monitoring behavior [14].

#### Output control

Output control focuses employee behavior through goal setting [15]. This entails communicating standards and goals and then providing staff with discretion in methods used to pursue established targets [16].

#### Input control

Input control equips the workplace with employees who have the right skills and abilities to do their job effectively. It includes a focus on the socialization of employees to the values of the organization as well as significant investments in recruitment and training of staff [12].

There are differences across studies as to the practices that constitute control or commitment-based HRM as well as differences in terminology. A comparison of these approaches and anticipated outcomes is provided in Table 1.

**Table 1:** A comparison of commitment-based approach and control-based approach to HRM

HRM practice	Commitment-based approach	Input control	Output control	Behavioral control
Recruitment/selection	• Selection to fit organization over job-specific requirements	• Rigorous selection to job and organization fit	• Selection of well-qualified staff	• Selection of staff to fit job/task requirements
Training and development	• Extensive training • Long-term investment in training	• Training plays the joint role of ensuring employees have the requisite abilities to do the job now and in the future as well as an understanding of the values and goals of the organization	• Ongoing firm-specific training and development	• Limited training or task-specific training
Performance review/appraisal	• Development oriented • Managers who facilitate rather than supervise	• The focus is on developing individuals who are intrinsically dedicated to the organization	• Supervisors set targets for employees to pursue • Information on results is readily available allowing employees to make their own judgments without supervisor intervention. • Employees have discretion in the method they use to accomplish established goals • Performance review is based on the results achieved	• Appraisal is based on supervisor close monitoring of employee behavior • Responsibilities are standardized and imposed top-down with an overriding concern for employees complying with procedures and methods and not results obtained • Feedback is used as a remedial tool
Reward and recognition	• Market compensation or above, aligned with team and organizational goals • Reinforcement of team achievements	• Limited focus on external inducements • Focus on the cultivation of intrinsic rewards	• Rewards are closely linked to performance outcomes	• Low wages and benefits
Predicted outcomes	• Committed and engaged workforce, high trust, cooperation, and positive social climate	• Cooperation, intrinsic dedication to the organization, and high levels of commitment and motivation	• Goal accomplishment as a means for achieving organizational effectiveness and performance	• Increased efficiency, reliability, and quality • Reduced direct labor costs and variation • Minimize cost

Adapted from [11, 13, 16, 26–28]

The comparison provided in Table 1 suggests that commitment and control are not necessarily at different ends of a continuum or an “either or” approach as suggested by Arthur [17]. Some aspects of input and output control are in line with a commitment-based HRM approach. Further, in the healthcare sector, a central goal is to minimize costs and maximize efficiency (as characterized by behavioral control) as well as build a committed and engaged workforce as predicted by commitment-based HRM.

Given the multi-faceted hospital environment, we draw on Snell’s control theory [12] and commitment-based HRM to explore how the approach to HRM in hospitals is implemented across four key functional areas, recruitment, performance appraisal, training, and reward and recognition, and the impact on the job attitudes of staff and operational efficiency. We acknowledge that there is lack of consensus of what constitutes “HR practices”; however, these four HRM functions are the most commonly drawn on in the HR literature that seeks to better understand commitment and motivation in employees [18].

*Research question one: How is HRM implemented in hospitals across different professional groups (physicians, nurses, allied health workers)*?

*Research question two: How is the HRM approach adopted by hospitals perceived, and how do these perceptions affect the job attitudes of staff and hospital operational efficiency*?

### Implementing HR in hospitals

The literature has portrayed line managers as the actors that translate intended HR practices into actual HR practices [19]. Australia now requires healthcare managers to assume oversight of multiple line management tasks including financial, operational, and HRM activities at the same time as ensuring patients receive high quality care. As such, dual management-clinical responsibilities are in place.^4^ One of the challenges for healthcare managers when implementing HR activities is the influence of social structures. For instance, the strategic direction of healthcare is largely determined by government agencies, which subsequently influence the organizational structure, policies, and rules in hospitals, which in turn dictate expectations of organizational members [20]. We now turn our attention to role theory to understand the complexity of the healthcare sector and the impact on HR.

Role theory is a framework that defines how individuals behave in social situations and how these behaviors are perceived [21]. Roles in organizations are assumed to be generated by normative expectations [22]. In healthcare, norms vary among employee groups and reflect both the official demands of hospitals, governing bodies of the profession, and the values associated with professional commitment. Given the varying norms of different stakeholders, healthcare managers can be subjected to role conflicts in which they must contend with antithetical norms [20]. For instance, in the context of budgetary constraints, healthcare managers may be pressured by executives to utilize a cost-effective approach to patient treatment instead of the best treatment which could lead to role conflict as most healthcare managers cite their primary role is to do whatever is possible to support people becoming healthy [23] or improve the quality of life of patients and their families who face life-threatening illness. Such role conflicts often produce stress, strain, and dissatisfaction [24].

Role conflict is also in place in regard to the practice of HR. Each healthcare professional group’s qualifications are determined by tertiary institutions and/or government registration. Medical and nursing colleges also play a part in determining a variety of HR obligations such as training requirements, standards for hiring, and evaluation of performance. The prevalence of multiple subsystems interacting with hospital preferences and professional groups is likely to create complexity for healthcare managers who are responsible for implementing HR, especially since people may be involved in various subsystems simultaneously [20]. For example, a healthcare manager needs to comply with training systems imposed by tertiary institutions and medical authorities at the same time as manage the government budgetary system and hospital resource system. As such, it is crucial to have an in-depth understanding of how healthcare workers negotiate different priorities and boundaries between these subsystems.

*Research question three: How do healthcare managers negotiate different priorities and boundaries between healthcare subsystems and HR practices*?

## Methods

### Sample characteristics

The multi-level research design was deemed important because of the nested hierarchical environment within hospitals. All data in the study was collected using in-depth interviews in a three-phase research design. Table 2 summarizes the professional groupings of the sample. Our interview protocol covered two general themes: (i) interviewees’ experience in regard to implementation of HRM practices and (ii) the consequences of these practices on job attitudes and work outcomes. We began the study by interviewing executives, healthcare managers, and employees as they were deemed to be a user or “customer” of HR activities in hospitals. The preliminary findings from the first phase of interviews (*n* = 7) suggested that there were differences across professional groups (doctors, nurses, and allied health workers) for each HR practice. Following this finding, we embarked on a second phase of data collection (*n* = 14) to understand why HR was implemented in hospitals the way it was. Findings from this second phase evolved into a final third phase of data collection (*n* = 13) to investigate the implications of HR on job attitudes of staff and hospital operational efficiency. Throughout our data analysis, member checks and peer examination strategies were used to reduce misinterpretation error and increase the credibility (internal validity) and dependability (reliability) of the findings [25]. The average duration of each interview was 38 min.

**Table 2:** Sample characteristics

Hospital executives total	6
Healthcare managers total • Physicians • Nurses • Allied health workers	12 4 5 3
Staff total • Physicians • Nurses • Allied health workers	16 6 5 5
Total interviews	34

The interviews were analyzed using NVivo and included open coding, axial coding, and theoretical coding, which enabled the examination of a broad range of interconnected processes and helped to link different causes and actions to specific outcomes. Our data analysis reached theoretical saturation after 13 iterations.

## Results and discussion

### The approach to HRM adopted

Using Snell’s control framework [12] and the commitment-based framework outlined in Table 1, our findings revealed a predominance of a control-based approach to people management; however, different forms of control were implemented across professional groups.

#### Recruitment: behavioral control

Lengthy recruitment practices were in place which required the completion of volumes of paperwork and multiple approvals. There was no managerial discretion to fast track decisions. Healthcare managers across the three professional groups found the recruitment process “very difficult and bureaucratized” (ID01) and “astoundingly cumbersome” (ID02).

#### Performance appraisal: output and behavioral controls

At the executive level, output control was primarily adopted across most HR practices. For example, executive appraisal includes a set of key performance indicators directly aligned with a hospital’s strategic plan. Executives did not have authority to develop objectives because government determined priorities, but they had discretion surrounding the means to achieve goals.

Hospitals showed marked differences with lower level employees whose performance was primarily managed through behavior control. Physician and nursing managers (who reported to executives) were evaluated using stringent monitoring mechanisms and standardized operating procedures without any known relationship with the hospital’s strategic plan. A nursing manager (ID06) explained she had to follow a strict routine to meet and abide by established procedures to ensure patients were discharged before 11 am. Such adherence to established protocols formed the major part of performance evaluation.

Appraisal among staff was commonly deemed as a form filling activity and not always conducted except in instances where hospital outcomes were not achieved (e.g., low levels of patient satisfaction on a ward).

#### Training: control and some evidence of commitment-based HRM

Behavior control was the dominant approach for decisions around training and development for nursing and allied health staff. Training was well documented, monitored, and audited. For nurses, mandatory training was compulsory for registration. Minimal non-mandatory training was available. Similarly, ongoing behavioral control and lengthy approval processes left little discretion for managers to make decisions or leverage HR to motivate staff. One allied healthcare manager (ID13) reported a “typical” multi-level approval process that extended past an actual conference date, resulting in non-attendance.

We found a mix between commitment-based HRM, behavior, output, and input controls in regard to the management of physicians. The HR approach adopted was determined according to the rank and influence of an individual physician. For junior doctors, training was prescribed by medical colleges and recorded by the physician manager. While such practices support behavior control, the development of specialized skills and knowledge was undertaken as a prerequisite to relinquish control or move from behavior to output control in the future.

In contrast to junior doctors, the approach adopted for senior physicians that reported to hospital executives was less rigid. As physicians progressed to higher levels, they were given discretion to determine appropriate development opportunities, which shifted from knowledge attainment to knowledge exploration, i.e., attending conferences and participating in research projects.

#### Reward and recognition: behavioral and output controls

Our findings revealed that there were two major forms of control used in reward and recognition practice. First, for nurses and allied health employees, there were strict rules that governed the administration of formal reward and recognition processes. For instance, in one large hospital’s reward program, staff could apply or be nominated by their peers for an excellence award. However, staff viewed the extensive nomination process as a heavy administrative task where the time and effort spent on applying for an award outweighed the benefits. The prevalence of behavior control in regard to rewards and recognition further embedded a bureaucratic culture leading to an inability of these schemes to be used by managers to motivate and improve morale. Second, in contrast, physician managers had some discretion to authorize non-mandatory training such as conference attendance, which was regarded as a form of reward.

### Negotiating different priorities and boundaries across subsystems

We found that hospitals did not have discretion to determine the HR approach adopted or the forms of control used. Instead, there were other demands influencing choice, such as the pressure to support government cost-cutting reforms and the need to comply with accreditation processes established by medical boards and colleges.

We noted earlier that the majority of healthcare managers were required to adopt a behavioral control perspective in regard to recruitment, professional development, and reward/recognition practices. Further analysis revealed that this inhibited healthcare managers’ ability to leverage HR practices as an employee engagement tool. Several physician managers explained how their role had evolved into a financial manager, and this created conflict with their own desire to adopt a stronger commitment-based HRM position to manage staff. We found that healthcare managers experienced tension juggling financial, people, and clinical responsibilities. One example indicative of similar experiences relayed during the interviews was shared by an allied health manager (ID23) who explained that she wanted to support one staff member’s professional development to undertake a course relevant to their job; however, clinical demands meant a replacement was required to cope with patient needs during the absence. This resource need and the cost of the training projected a budget shortfall. The strain between the financial, human resource, and clinical subsystems ultimately resulted in the staff member not being able to participate in the professional development activity, leading to frustration for the manager and employee.

Another subsystem healthcare managers had to comply with was mandatory training of healthcare workers as determined by professional groups, tertiary institutions, and/or government bodies. This restricted all discretion of healthcare managers as the structure and timing of the training was often prescribed and contributed to competency assessment of hospitals by medical authorities.

### How HRM is perceived

Employees perceived the HRM function as non-value adding and a major source of bureaucratization. Centralized HRM processes and practices were seen as inefficient and poor quality. Healthcare managers were frustrated with the HRM function due to a lack of responsiveness and support. HRM professionals were perceived as (i) administrative generalists that manage leave and payroll, (ii) transactional and non-strategic, and (iii) punitive and destructive.

In order to obtain a balanced understanding of HRM in hospitals, we also asked HR specialists about their perceptions. HRM professionals in hospitals agreed with the views shared by employees and reflected that their roles largely focused on transactional tasks such as data collection, grievances, hiring inquiries, payroll, and training administration. HRM specialists aspired to more strategic business partner roles that included the formulation of vision and strategic plans; however, limited resources and inadequate technology systems prevented HRM staff operating at a strategic level. Further, a lack of credibility due to a poor track record across multiple HRM functions inhibited HRM staff being able to influence such a change.

### Negative job attitudes and inefficient operations

The overuse of behavioral control and at times inappropriate use of behavioral control resulted in feelings of frustration, resentment, discontent, indifference, and negative perceptions of senior executives and hospitals. This was consistent across professional groups. An example of this can be seen in regard to recruitment. When recruitment paperwork was correctly completed and interviews carried out, approval could be revoked and healthcare managers left unsure if the preferred candidate would ultimately be employed. In many cases, by the time the necessary procedures were carried out, candidates were employed elsewhere. One of the nursing managers (ID30) expressed her frustration that such inefficient processes led to a waste of resources and time, and her suspicion was that such delays were deliberate to save on staff costs. This view was supported by physicians. Managers also perceived their authority as a leader was undermined because of an inability to communicate to staff the outcome of recruitment. Managers and staff spoke of their annoyance having to complete excessive paperwork all over again for a new candidate or for one that was already employed in a partner government hospital. The lengthy process also exerted considerable pressure on existing staff that were required to assume additional responsibilities or shifts until the vacant position was filled.

Such inefficient processes in an essential HR function also distracted managers’ attention from patient-centered care. ID02 described her role as a nursing manager: “This job has changed. The management role now is just astounding. I work 10 hours a day. I start early and I finish late and I pretty much sit at this desk all day. I can’t think of the last time I really saw a patient…there is no secretarial support, so I’m answering the phone all day…managing pay systems, recruiting systems.”

A surgical physician manager provided additional insights on how the outcomes of behavioral control adversely affect job attitudes: “it breeds a level (of) malcontent within junior nurses…. I go into my operating department and there will be between six and fifteen nurses who ring in sick. I am certain that they are not all physically unwell” (ID24).

Our analysis further revealed that an overarching hospital objective and priority to cut costs led to the development of behavioral controls in the form of overly complex, duplicative, and bureaucratic processes. For example, the stringent approval process for professional development was aimed at reducing expenditure while the recruitment processes described above were established as a means to reduce costs associated with the clinical budget.

However, our findings also revealed that staff perceived the outcome of cost savings to be questionable due to the inefficiency of the process. Healthcare managers and staff believed that cost-cutting measures and the control-based orientation to people management were detrimental to patient care. For example, ID32 an allied healthcare employee who works in mental health explained how executives’ focus on costs had driven practices to discharge patients prematurely. However, she believed the opposite effect occurred because such decisions ultimately cost more as patients required welfare assistance following discharge. In most cases, such patients were readmitted into the system because they were not well enough to be discharged in the first place. Hence, the hospital served as a “revolving door” for the same patients, though on paper, the length of stay and other key budgetary indicators highlighted executives’ superior performance in meeting KPIs.

## Conclusions

Our research provides insights into the HR approach adopted by Australian hospitals and the implications on employee attitudes and hospital operations. Using Snell’s control framework [12], we found that behavioral control was the predominant form of control used to manage nurses, allied health workers, and junior doctors. However, we found a mix between behavior, output, and input controls as well as elements of commitment-based HRM to manage senior physicians.

We suggest that hospitals reduce the profusion of behavior control and increase the levels of input and output controls in the management of people. We do not support popular views that hospitals should implement a commitment-based HRM strategy as these recommendations are overly optimistic and based on private sector best practice and inappropriate considering the subsystems and influence of external bodies affecting healthcare. Instead, we suggest that the benefits of a commitment-based approach can be built through input and output controls, which are likely to cultivate employee engagement.

We found that hospitals used rules to promote conformity with established procedures; the overuse and at times inappropriate use of behavior controls restricted healthcare managers’ ability to motivate and engage their staff. Excessive use of behavior control helped to realize short-term cost-cutting goals; however, this often led to operational inefficiencies. We also observed low levels of investment in people and a concentration on transactional HR activities which led to negative job attitudes such as low morale and frustration among healthcare professionals.

Our findings illustrate that different regulatory “powers” operating in hospitals further complicate the HR practices of training, performance, and reward. Unlike typical corporations, hospitals are unique because the employees are not fully regulated by the hospital. Registration and training requirements of healthcare workers are determined by tertiary institutions, government bodies, and medical and nursing colleges—completely outside the influence of the HR function and hospital executives.

Our findings suggest that hospitals serve as a “facility” where professionals congregate to deliver their services and where employees view the hospital and HR function as entities that need to be tolerated in order to practice medicine. Hence, our study concurs with research that HRM research cannot be implemented in all contexts in the same way. The complexity of HRM in healthcare involves a consideration of external factors and structures within the healthcare system. In particular, our results illustrate how the different regulatory authorities and professional bodies constrain executives and HR practitioners’ ability to effectively carry out strategic HRM. The differing strategies of the various authorities leave little room to determine how practices such as reward and training are implemented. We also found that healthcare managers experience challenges and conflict in their role because they strive to support their staff by providing more professional development but this creates conflict meeting financial requirements.

Our study provides insights on why HR is perceived to contribute limited value in hospitals. Healthcare workers and managers perceive HR activities as contributing to bureaucracy and are largely administrative in orientation. Subsequently, poor perceptions result in HR being overlooked as a vehicle to address the strategic challenges required of health reform and to build an engaged workforce. Yet, given the challenges facing the sector, the “strategic partner” and “change agent” roles where HR professionals partner with healthcare managers and executives to achieve goals through culture change seem to be exactly what is needed to deliver necessary healthcare reforms. We hope that this study may serve as a launching pad for HR and those responsible for quality clinical outcomes to collaborate to work on the various strategic projects that heavily rely on healthcare professionals to succeed.

## Footnotes

1 Hospital executives are responsible for planning, strategizing, and the coordination of the health services of a hospital. Executives analyze the effectiveness of health services of various departments and work to reach financial goals and maintain budgets. While many executives have a health-related professional background (e.g., as a physician or nurse), this is not always the case.

2 A healthcare manager supervises healthcare staff, helps implement policy and procedure recommendations, and handles numerous financial, operational, and HRM administrative duties. Duties are mainly undertaken in the context of a ward or specialist unit (e.g., intensive care). All healthcare managers have clinical responsibilities in the department or unit they lead. A “nursing” healthcare manager will delegate patient assignments, contact physicians for treatment orders, and solve problems associated with staff or patients. A “physician” healthcare manager supervises junior and intern doctors, coordinates with consulting physicians, and usually overseas a speciality department. An “allied health” manager coordinates a multidisciplinary healthcare team (e.g., social workers, physiotherapists, or occupational therapists) that works in speciality departments or wards.

3 Employees are clinical healthcare workers without management responsibilities. Employees could be nurses, allied health workers (social workers, physiotherapists, or occupational therapists), or physicians.

4 While this dual management-clinical model has been adopted in Australia and other countries, it is not universally employed.
